# Bilateral auricular nodules: A peculiar presentation of systemic mantle cell lymphoma

**DOI:** 10.1016/j.jdcr.2021.05.014

**Published:** 2021-06-02

**Authors:** Mike Fritz, Martin Dittmer, Daniel Tinker, Kristin Smith, Katherine Robbins, Linda Goldenberg, Mark Fesler, M. Yadira Hurley

**Affiliations:** a University of Kentucky College of Medicine, Lexington, Kentucky; b Department of Dermatology, Saint Louis University, St. Louis, Missouri; c Department of Pathology, Saint Louis University, St. Louis, Missouri; d Division of Hematology, Oncology, and Cellular Therapy, Department of Internal Medicine, Saint Louis University, St. Louis, Missouri

**Keywords:** auricular swelling, B-cell, cancer, cutaneous manifestation, cutaneous mantle cell lymphoma, extranodal sites, immunohistochemistry, lymphoma, mantle cell lymphoma, non-Hodgkin lymphoma, rituximab, TP53, IHC, immunohistochemistry, MCL, mantle cell lymphoma, NHL, non-Hodgkin Lymphoma

## Introduction

Mantle cell lymphoma (MCL), a rare malignancy of B cells accounting for 6% of all non-Hodgkin lymphomas (NHL),^1^ arises due to t(11;14)(q13;23) translocation of mature B lymphocytes in the mantle zone of lymph node follicles. This translocation results in the overexpression of cell cycle regulator cyclin D1 and a heterogeneous but often aggressive NHL subtype. MCL is characterized by CD5^+^, CD20^+^, CD79a^+^, and cyclin D1^+^ tumor cells.^2^

MCL generally presents in the fifth or sixth decade, affects men more than women, and presents at an advanced stage (stage III or IV).^3^ Patients present with classic “B” symptoms, diffuse lymphadenopathy, or symptoms specific to the extranodal site of involvement. MCL has a propensity to involve the gastrointestinal tract, spleen, liver, peripheral blood, and bone marrow.^3^ Cutaneous metastasis as the presenting symptom of MCL is an extremely rare entity. Herein, we describe the second reported case of systemic MCL presenting as violaceous bilateral ear swelling with papules and nodules with an otherwise negative review of systems.

## Case report

**Fig 1:**
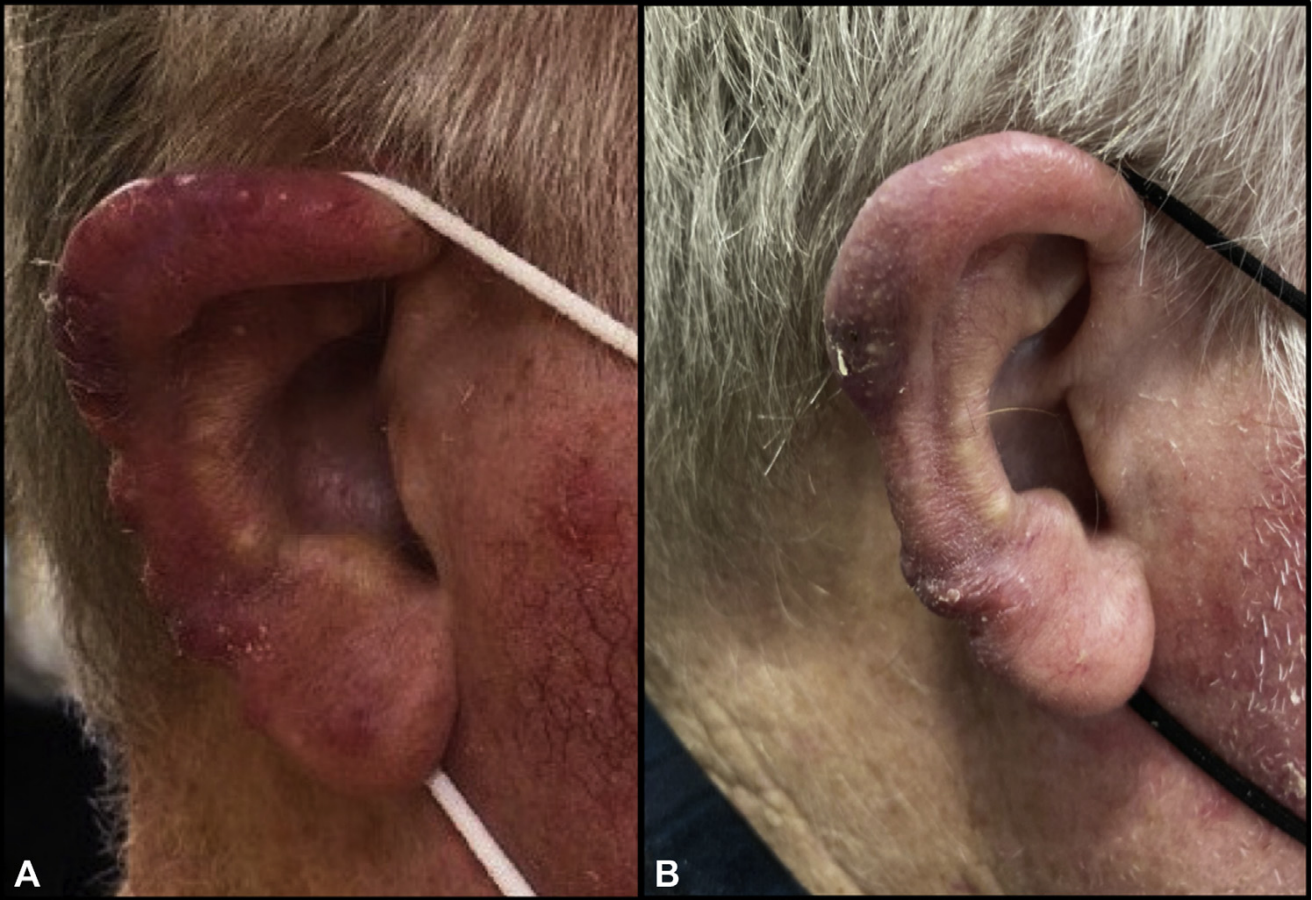
Mantle cell lymphoma of the right ear presenting as auricular edema and papulonodules, some with overlying hemorrhagic scale crust, involving the pinnae, helices, antihelices, and lobules. The condition notably improved (**B**) after rituximab therapy compared with (**A**) that during the initial presentation.

**Fig 2:**
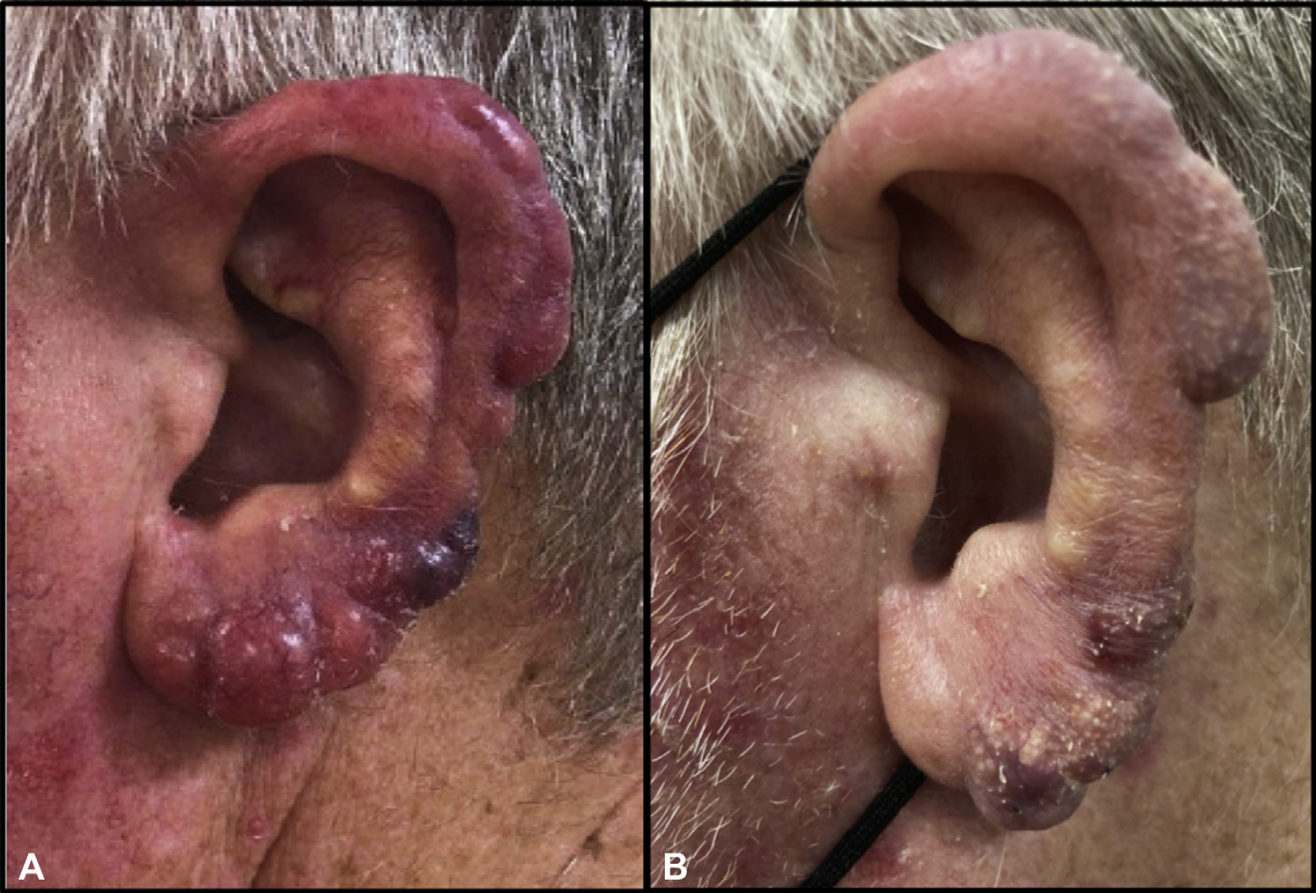
Mantle cell lymphoma of the left ear presenting as bilateral auricular edema and papulonodules, some with overlying hemorrhagic scale crust, involving the pinnae, helices, antihelices, and lobules. The condition notably improved (**B**) after 8 rituximab infusions compared with (**A**) that during the initial presentation.

A 69-year-old Caucasian man with no significant past medical history presented to a dermatologist with a 6-month history of painless swelling, redness, and bumps on the lateral surface of both ears. He denied fever, chills, night sweats, or unintentional weight loss. He also denied nausea, vomiting, or abdominal pain and had no history of being immunocompromised. Physical examination of the middle-aged man revealed 10 to 20 dark red-to-violaceous, scalloped papulonodules involving the lateral surface of his ears (Figs 1 and 2). The findings were associated with a palpable, firm, nontender, and mobile 1-cm lymph node in the left superior cervical chain.

**Fig 3:**
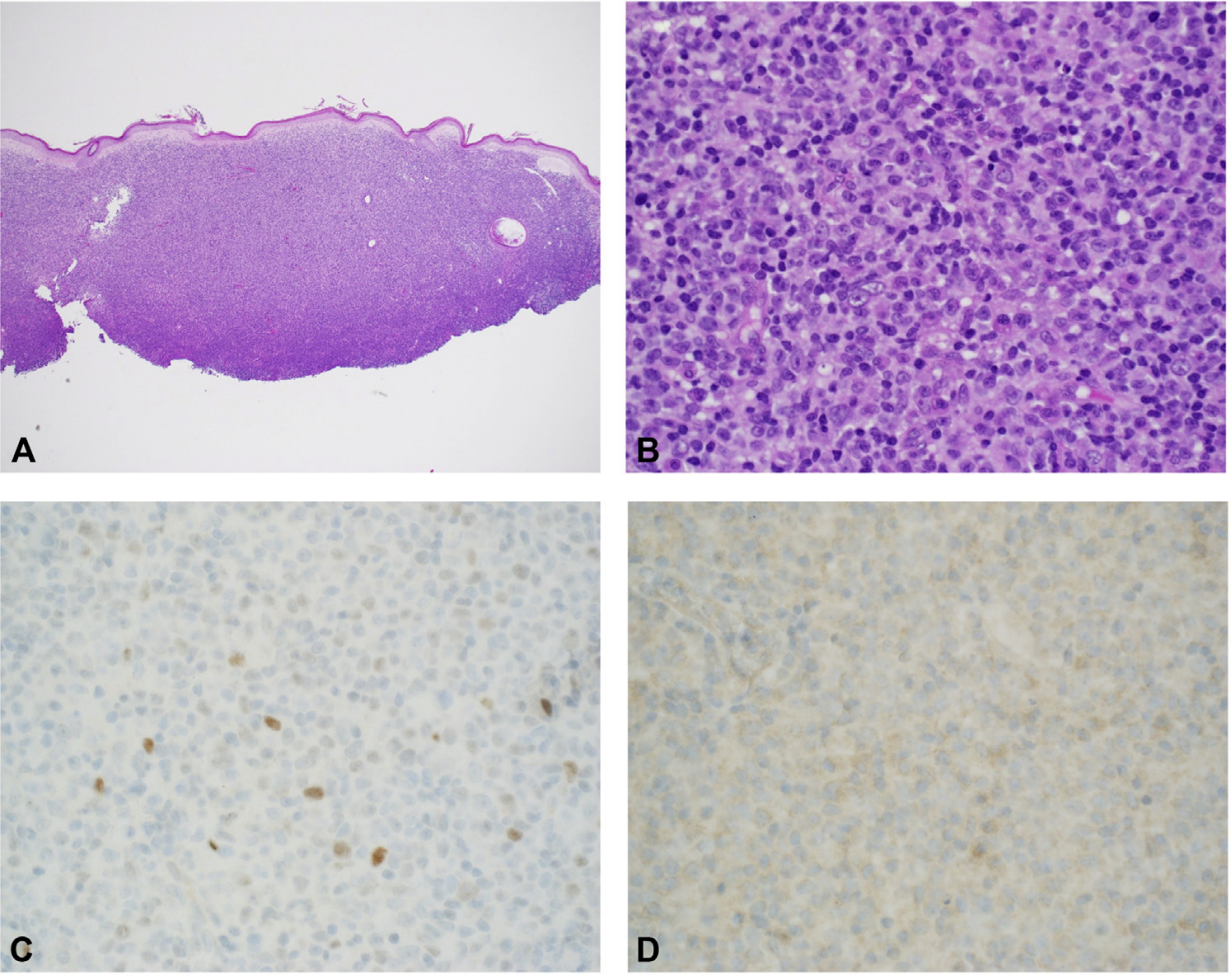
**A**, Cutaneous mantle cell lymphoma photomicrographs showed a dense lymphocytic infiltrate in the dermis with overlying grenz zone. **B**, High magnification demonstrated small- to intermediate-sized lymphocytes, many with irregular nuclear contours and variable nucleoli. **C**, p53 immunohistochemistry from the skin biopsy highlighted 5% to 10% of tumoral cells that were consistent with the low expression of p53. **D**, The cells were also SOX11^−^. (**A** and **B**, Hematoxylin-eosin stain; **C**, p53 immunohistochemical stain; **D**, SOX11 immunohistochemical stain; original magnifications: **A**, ′4; **B**, ′60.)

Shave biopsies from both ears were obtained at the visit. Complete blood cell count, comprehensive metabolic panel, serum lactate dehydrogenase, positron emission tomography/computed tomography, and flow cytometry of peripheral blood were ordered. Both biopsies revealed an atypical dermal B-cell lymphoid infiltrate of small- to medium-sized cells expressing Pax-5, diminished Bcl-2, and CD20. CD21 did not reveal germinal centers or a dendritic cell meshwork. Bcl-6 was negative, and Ki-67 appeared low within the atypical infiltrate. The examination of peripheral blood revealed absolute lymphocytosis with a lymphocyte count of 5.34 × 10^3^ cells/μL, and flow cytometric analysis demonstrated a CD5^+^, CD23^−^ mature B-cell lymphoma, consistent with MCL. Staging positron emission tomography/computed tomography showed fluorodeoxyglucose avidity of the bilateral pinnae, liver, spleen, and bilateral cervical and axillary lymph nodes. Left axillary lymph node excision confirmed aberrant CD5 positivity, diffuse cyclin D1 expression of B cells, and Ki-67 appeared positive in 5% to 15% of the cells, confirming the diagnosis of systemic MCL. Cutaneous biopsies showed a dense dermal lymphocytic infiltrate with irregular nuclear contours (Fig 3, *A* and *B*). SOX11 stained very rare nuclei and p53 highlighted 5% to 10% of tumor cells (Fig 3, *C* and *D*). *TP53* mutation analysis was negative, and chromosome analysis was not reported because of the absence of metaphase cells. The patient was referred to oncology and initiated on rituximab treatment, after which his cutaneous lesions began to improve.

## Discussion

MCL is a rare form of NHL, comprising only 6% of all NHL.^1^ The characteristic t(11; 14)(q13; 23) translocation leads to aggressive lymphoma, and most patients are diagnosed with stage III or IV disease.^3^ Cutaneous involvement in paraneoplastic syndromes, such as dermatomyositis, are more commonly observed in MCL than cutaneous metastasis of the systemic disease.

This is just the second reported case of systemic MCL that presented with bilateral auricular involvement.^4^ Such a distribution, in the absence of systemic signs or symptoms, may masquerade as an inflammatory dermatosis, including cutaneous lymphoid hyperplasia or an indolent lymphoma. In most cases, the demonstration of cyclin D1 overexpression by immunohistochemistry (IHC) establishes the diagnosis in the setting of an otherwise supportive immunophenotype; however, a select immunohistochemical panel, including CD5, serves as the first step in screening for this rare entity.

In recent years, *TP53* mutational status has garnered recognition as a useful prognostic factor in systemic MCL. *TP53* mutated MCL is associated with resistance to traditional chemotherapy and increased relapse rates.^5^^,^^6^ Although such cases have also been linked to blastoid cytology and high proliferation indices, morphology alone does not predict *TP53* status. Thus, efficient proxies are needed when molecular analysis is unavailable or impractical. The demonstration of p53 overexpression (>30% of nuclei) by IHC in tissue microarray has been advanced as a reliable surrogate for *TP53* missense mutations in MCL, with 82% sensitivity and 100% specificity reported.^5^ It is hypothesized that the mutant p53 protein is relatively stable compared with the wild-type protein, leading to intracellular accumulation demonstrable by IHC. Importantly, complete loss of p53 expression by IHC carries significant prognostic relevance, which exceeds any karyotype, fluorescence in situ hybridization, or immunohistochemical analysis.^6^^,^^7^ SOX11, initially championed in the diagnosis of cyclin D1^−^ MCL, has likewise been investigated as an MCL oncogene and prognostic marker, with mixed results. The absence of SOX11 (<10% nuclear staining) has been linked to leukemic nonnodal disease (with relatively favorable prognosis) as well as to p53 overexpression in a small series.^8^ Few groups have examined MCL prognostic markers in cutaneous specimens.^9^^,^^10^ Our case showed congruence between low p53 expression in skin and negative mutational analysis of nodal tissue. Additionally, SOX11 was negative, in contrast to 11 of the 11 cutaneous cases staining >25% of cells in the series by Wehkamp et al.^9^

We report a highly unusual clinical presentation of an exceedingly rare phenomenon: systemic MCL presenting on the lateral surface of both ears with an otherwise negative review of systems. Our case underscores the myriad presentations that cutaneous MCL may assume and emphasizes the importance of dermatologists possessing low thresholds to biopsy suspiciously appearing dermal nodules and to workup for systemic involvement. Readers are also alerted to relevant trends in prognostic stratification of MCL using IHC of hematopathology specimens. Familiarity with such markers and their integration into clinical practice is of paramount importance in the diagnosis and prognosis of patients with cutaneous MCL. Our case is consistent with prior studies showing agreement between p53 IHC, mutational status, and response to treatment but emphasizes a need for further vetting of p53 and SOX11 IHC in cutaneous specimens. Based on our experience, it is also ideal for institutions to support multidisciplinary cutaneous lymphoma clinics, where the clinic structure facilitates the most efficient communication between providers and the patient.

## Conflicts of interest

None disclosed.
